# Quantification of mutant alleles in circulating tumor DNA can predict survival in lung cancer

**DOI:** 10.18632/oncotarget.8021

**Published:** 2016-03-10

**Authors:** Xue Yang, Minglei Zhuo, Xin Ye, Hua Bai, Zhijie Wang, Yun Sun, Jun Zhao, Tongtong An, Jianchun Duan, Meina Wu, Jie Wang

**Affiliations:** ^1^ Department of Thoracic Medical Oncology, Key Laboratory of Carcinogenesis and Translational Research (Ministry of Education), Peking University Cancer Hospital and Institute, Beijing, China; ^2^ Asia and Emerging Markets Innovative Medicine of AstraZeneca R & D, Shanghai, China

**Keywords:** non-small-cell lung cancer, epidermal growth factor receptor, circulating tumor DNA, droplet digital PCR, next-generation sequencing

## Abstract

**Purpose:**

We aimed to investigate the feasibility of droplet digital PCR (ddPCR) for the quantitative and dynamic detection of EGFR mutations and next generation sequencing (NGS) for screening EGFR-tyrosine kinase inhibitors (EGFR-TKIs) resistance-relevant mutations in circulating tumor DNA (ctDNA) from advanced lung adenocarcinoma (ADC) patients.

**Results:**

Detection limit of EGFR mutation in ctDNA by ddPCR was 0.04%. Taking the EGFR mutation in tumor tissue as the golden standard, the concordance of EGFR mutations detected in ctDNA was 74% (54/73). Patients with EGFR mutation in ctDNA (*n* = 54) superior progression-free survival (PFS, median, 12.6 vs. 6.7 months, *P* < 0.001) and overall survival (OS, median, 35.6 vs. 23.8 months, *P* = 0.028) compared to those with EGFR wild type in ctDNA (*n* = 19). Patients with high EGFR-mutated abundance in ctDNA (> 5.15%) showed better PFS compared to those with low EGFR mutated abundance (≤ 5.15%) (PFS, median, 15.4 vs. 11.1 months, *P* = 0.021). NGS results showed that 66.6% (8/12) total mutational copy number were elevated and 76.5% (26/34) mutual mutation frequency increased after disease progression.

**Methods:**

Seventy-three advanced ADC patients with tumor tissues carrying EGFR mutations and their matched pre- and post-EGFR-TKIs plasma samples were enrolled in this study. Absolute quantities of plasma EGFR mutant and wild-type alleles were measured by ddPCR. Multi-genes testing was performed using NGS in 12 patients.

**Conclusions:**

Dynamic and quantitative analysis of EGFR mutation in ctDNA could guide personalized therapy for advanced ADC. NGS shows good performance in multiple genes testing especially novel and uncommon genes.

## INTRODUCTION

Great advances have been made in the treatment of non-small cell lung cancer (NSCLC) over the past 40 years. The discovery of oncogenic drivers such as EGFR (epidermal growth factor receptor) has enabled a new and increasingly efficacious phase in cancer treatment [1–4]. Nevertheless, almost 50% patients develop resistance to EGFR-TKIs in 9–12 months [5–10]. Clinical monitoring molecular resistance through second biopsy of tumor tissues can be challenging. Moreover, owing to spatial and temporal heterogeneity, molecular detection methods that use initial tissue samples are not appropriate for therapeutic guidance throughout the entire process of treatment, especially after disease progression. Therefore, methods for detecting mutations have been investigated in other specimens that can be acquired easily and dynamically; examples include the use of specimens like plasma [11–15], pleural fluid [15–17], and sputum [18, 19], among which plasma testing has been identified as the most promising tool in EGFR mutation analysis [11, 20].

Previous studies have shown the feasibility of investigating EGFR mutation status in ctDNA [21–24]. However, most of these prior studies did not present methods for the dynamic and quantitative evaluation of ctDNA. They simply performed qualitative detection of EGFR mutations in plasma. To date, reported quantitative detection methods for evaluating EGFR mutation status include BEAMing (beads, emulsion, amplification, magnetics), ddPCR, and NGS-based methods. Each of these approaches has their own advantages and disadvantages [25–30]. Here, we chose and evaluated the performance of two methods, ddPCR and NGS, to address the commonly encountered problems of low sensitivity and complex multi-gene testing.

This study aimed to assess the concordance and feasibility of ddPCR and NGS for the detection of mutations in plasma samples. Moreover, we also evaluated the clinical outcomes according to the quantity of EGFR mutations in advanced NSCLC.

## RESULTS

### Patient characteristics

Seventy-three patients from Peking University Cancer Hospital diagnosed with lung adenocarcinoma, including 29 male and 44 female patients, were enrolled in the study. Fifty-three of the patients had never smoked (72.6%, 53/73) and 20 were former/current smokers. All of the patients carried EGFR mutations in their TKI-naïve tissue samples: there were 41 cases for exon 19 deletion alone, 30 cases for the L858R mutation alone, and 2 cases harboring concomitant exon 19-del and L858R mutations. Thirty-seven patients (51.0%, 37/73) received EGFR-TKIs as the first-line therapy, while the rest received EGFR-TKIs treatment as second (or beyond) line treatment. The patients’ clinical characteristics are listed in Table 1. Survival data were followed till the end of October 2014, and the median follow-up period was 23.8 months (range, 4.3 months–52.8 months). By the end of the last follow-up, sixty-seven patients experienced progressive disease (PD) and thirty-eight of the patients had died. The mortality rate was 52.1% (38/73).

**Table 1:** Patients’ clinical characteristics (*n* = 73)

Characteristics	No of Patients	% of patients
Age, years		
Median	58	
Range	34–89	
Gender		
Male	29	40
Female	44	60
Smoking status		
Never smoker	53	73
Former/current smoker	20	27
EGFR-TKIs treatment line		
First line	37	51
Second or beyond	36	49
EGFR-TKIs failure modes	*n* = 67	
Dramatic PD	23	34
Slow PD	44	66

EGFR: epithelial growth factor receptor; TKI: tyrosine kinase inhibitor; PD: progression of disease.

Matched plasma samples, both pre-EGFR-TKIs therapy and post-PD of EGFR-TKIs, were obtained form 67of 73 patients. The time interval from the diagnosis of PD to blood sampling for ddPCR was no more than four weeks, with no intervening chemotherapy. The matched plasma samples for the other 6 patients were obtained during treatment without disease progression.

### Evaluation of the consistency of activating EGFR mutations between TKI-naïve tissue and plasma DNA by ddPCR

Fifty-four of 73 patients were positive for EGFR mutations in *de novo* ctDNA (31 cases for exon 19 deletion, 23 cases for L858R). EGFR mutations in ctDNA were identified in 74% (54/73)of the patients that had documented EGFR mutations in their tumors. The median absolute and relative EGFR mutant allele quantities in TKI-naive plasma from 54 patients was 487 copies/reaction and 5.15% respectively. The response rates (RR) and disease control rates (DCR) were not significantly different between patients with EGFR mutant and wild-type alleles.

### Qualitative and quantitative analysis of EGFR mutations in *de novo* plasma by ddPCR predicted survival

OS_1_ was defined as the first day of the TKIs or chemotherapy until death from any cause or the date of the last follow-up. OS_2_ was defined as the time from disease progression after EGFR-TKIs therapy to death from any cause or the date of the last follow-up. OS_1_ represented the overall survival and OS_2_ stood for the post-TKIs survival.

**Figure 1:**
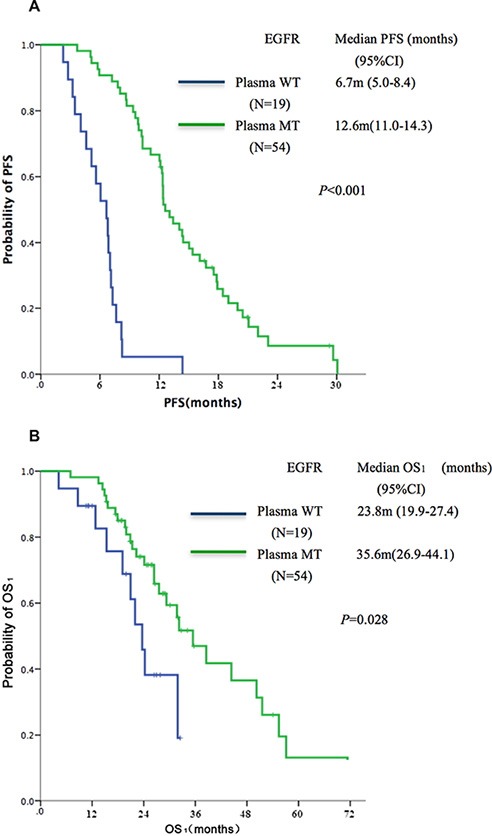
Kaplan-Meier curves of (A) PFS and (B) OS according to qualitative analysis of sensitive EGFR mutation (19del or L858R) in TKI-naive plasma samples identified by ddPCR (*n* = 73) MT: mutant type; WT: wild type.

According to the EGFR mutation status of ctDNA in TKI-naïve patients, all 73 patients were divided into two subgroups: a group that carried mutations in both specimens (T^+^/B^+^, *n* = 54), and a group that carried mutations only in tissues rather than in ctDNA (T^+^/B^−^, *n* = 19). The T^+^/B^+^ group showed superior PFS (median, 12.6 vs. 6.7 months, *P* < 0.001, Figure 1A) and OS_1_ (median, 35.6 vs. 23.8 months, *P* = 0.028) as compared to the T^+^/B^−^ group (Figure 1B).

**Figure 2:**
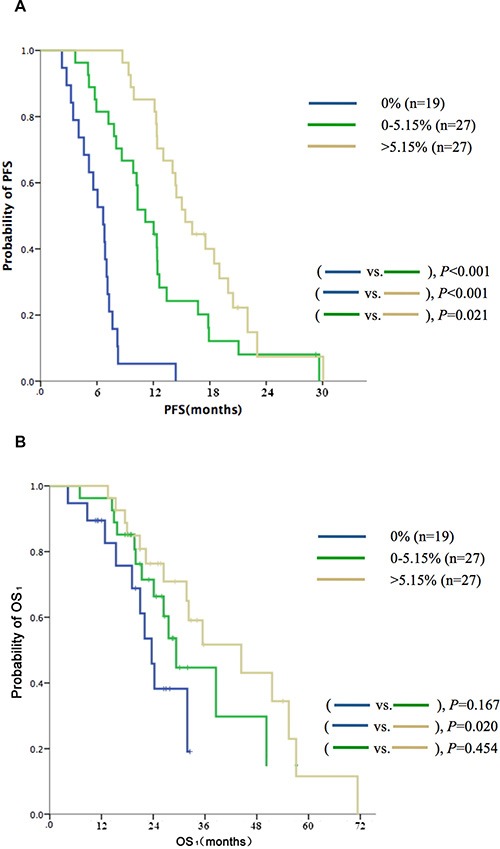
Kaplan-Meier curves of (A) PFS and (B) OS according to quantitative analysis of sensitive EGFR mutation (19DEL or L858R) in TKI-naive plasma samples identified by ddPCR (*n* = 73)

In addition to the qualitative analysis of EGFR mutations, quantitation of EGFR mutant alleles was also performed. In the cohort of 73 cases, the patients were subdivided into three groups based on the relative quantity of EGFR mutant alleles (median,5.15%) in TKI-naive plasma samples (high: > 5.15%, *n* = 27; low: ≤ 5.15%, *n* = 27; and nil: 0%, *n* = 19); the respective median PFS values were 15.4 vs. 11.1 vs. 6.7 months (*P* < 0.001, Figure 2A); the respective median OS_1_ values were 44.5 vs. 29.3 vs. 23.8 months (*P* = 0.072, Figure 2B). Selected characteristics of patients with different EGFR abundances are shown in Table 2.

**Table 2:** Selected characteristics of patients with different abundances of EGFR mutations (*n* = 54)

Variables	EGFR abundance				*P* value
	Low abundance (*N* = 27)		High abundance (*N* = 27)		
	*N*	%	*N*	%	
Age					*1.00*
< 65 y	21	77.8	21	77.8	
≥ 65 y	6	22.2	6	22.2	
Gender					*0.40*
Male	10	37.0	13	48.1	
Female	17	63.0	14	51.9	
Smoking History					*0.36*
Former smoker	6	22.2	9	33.3	
Never smoker	21	77.8	18	66.7	
EGFR-TKIs treatment line					*0.10*
First line	15	55.6	9	33.3	
Second or beyond	12	44.4	18	66.6	
DCR					*0.58*
PR	13	48.1	11	40.7	
SD	14	51.9	16	59.3	

Notes: factors were tested by χ^2^ test.

Abbreviations: EGFR, epithelial growth factor receptor; TKIs, tyrosine kinase inhibitors; DCR, disease control rate; PR, partial remission; SD, stable disease.

No correlation was found between post-PD EGFR mutation abundance and OS_2_ (median, 17.2 vs. 16.6 months, *P* = 0.247). No significant differences were found between the absolute quantity of in post-PD plasma samples.

### Dynamic change in the abundance of EGFR mutations was associated with survival

Analysis of the plasma DNA from the 67 patients with PD, 29 cases (43.3%, 29/67) showed decreasing EGFR mutation abundance following EGFR-TKIs treatment, 13 cases (19.4%, 13/67) kept the same EGFR mutation abundance following EGFR-TKIs treatment, and 25 (37.3%, 25/67) cases showed increasing EGFR abundance following EGFR-TKIs treatment.

**Figure 3:**
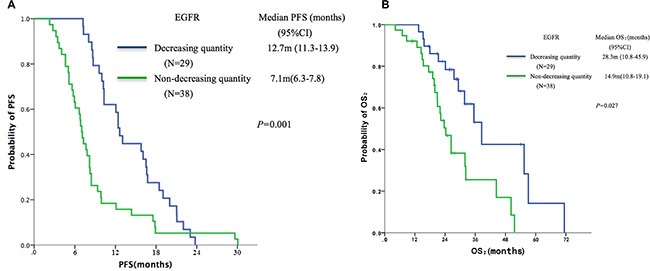
Kaplan-Meier curves of (A) PFS and (B) OS according to dynamic changes of EGFR abundance in plasma samples by identified ddPCR (*n* = 67)

The 67 patients with PD were divided, based on dynamic changes in EGFR mutation abundance in plasma DNA, into the ‘decreasing quantity group’ (*n* = 29) and the ‘non-decreasing quantity group’ (*n* = 38). Patients in the decreasing quantity group showed better PFS (median, 12.7 vs.7.1 months, *P* = 0.001, Figure 3A) and OS_2_ (median, 28.3 vs.14.9 months, *P* = 0.027, Figure 3B) as compared with the non-decreasing quantity group.

### Detection of T790M status in PD plasma samples using ddPCR

The T790M mutation was detected in 29 out of 67 (43.3%) patients when their disease progressed after EGFR-TKIs therapy. Fourteen of 29 T790M-positive patients expressed the elevation of EGFR mutated abundance after PD, while another 15 patients demonstrated the decline of EGFR mutated abundance after PD. No correlation was found between the T790M mutation and survival.

The potential relationships between clinical modes of EGFR-TKI failure (dramatic PD and slow PD) and T790M status were also analyzed. Dramatic PD was defined as rapid progression of multiple organs or target lesions or symptom deterioration after disease control lasting ≥ 3 months with EGFR-TKIs treatment. Slow PD was defined as gradual and local progression excluding dramatic PD [31]. There were 23 patients with dramatic PD modes and 44 patients with slow PD modes. No correlation was found between T790M mutation status and TKI-failure modes (*P* = 0.975).

### The relationship of ctDNA with metastasis site and previous treatment

Considering the potential effects of numbers of metastasis site (tumor burden) and previous treatment on ctDNA content, we investigated the relationships between these factors with ctDNA levels by Kruskal-Wallis Test. The *de novo* plasma samples had higher ctDNA levels than those obtained after front-line treatment (namely, EGFR-TKIs as first line versus non-first line, *P* = 0.025). However, no association was found between numbers of metastasis sites and ctDNA levels (*P* = 0.373). We put these two factors (metastasis site and previous treatment) into the Cox regression and found that EGFR mutation status of *de novo* plasma determined by ddPCR remained to be an independent predictor of PFS of EGFR-TKIs therapy (Table 3).

**Table 3:** Association between clinical features and PFS using multivariate analysis

		P	HR	95% CI
Previous treatment	EGFR-TKI first line	0.016	0.514	0.298–0.885
Number of metastatic sites	1	0.009		
	2	0.359	1.351	0.710–2.572
	3+	0.002	2.634	1.420–4.884
EGFR mutation status	Negative	0.000		
	Low abundance	0.000	0.122	0.057–0.258
	High abundance	0.000	0.066	0.030–0.149

HR: hazard ration; CI: confidence interval.

### Frequently mutated genes and altered pathways in ctDNA determined by NGS

To explore other resistance mechanisms beyond T790M, we randomly selected 12 matched plasma DNA pre-EGFR-TKIs and post-PD from 67 patients for deep sequencing. Of 12 patients, 10 had matched plasma samples both pre-EGFR-TKIs and post-PD, another two cases (cases 25 and 29) had multi-point plasma samples (Table 4). The total plasma sample number used for the NGS detection experiment was twenty-seven. The sensitivity of the deep sequencing protocol with target regions for 483 genes designed by Agilent SureSelect allowed for the detection of a mutant allele fraction of 5%, with a mean sequencing depth of 609 ×. For each patient, if the mutant fraction less than 5%, it would not be directly detected by the software. However, if other samples of the same patient harbored mutant fractions of more than 5%, then the lost information for a given sample could be retrieved.

**Table 4:** Patient characteristics and NGS monitoring results in 12 cases

Patient ID	Gender	Age	Histology	Smoking status	Tumor stage	Best response	PFS (months)	N. of Somatic SNV					N. of Somatic InDel				
								baseline	C3	C4	PD	PD6	baseline	C3	C4	PD	PD6
1	F	46	Adeno	N	IV	PR	19.4	8	-	-	10	-	0	-	-	0	-
4	M	80	Adeno	N	IV	PR	17.3	5	-	-	6	-	0	-	-	0	-
8	M	59	Adeno	N	IV	SD	18.2	4	-	-	5	-	2	-	-	2	-
10	F	53	Adeno	N	IV	SD	8.6	1	-	-	1	-	1	-	-	1	-
14	M	87	Adeno	Y	IV	SD	17.7	7	-	-	11	-	0	-	-	0	-
16	M	54	Adeno	Y	IV	SD	12.4	1	-	-	10	-	0	-	-	0	-
17	F	34	Adeno	N	IV	SD	3.3	4	-	-	6	-	1	-	-	2	-
23	M	73	Adeno	N	IV	SD	9.6	1	-	-	1	-	0	-	-	0	-
24	F	73	Adeno	N	IV	SD	8.5	4	-	-	5	-	2	-	-	2	-
25	F	65	Adeno	N	IV	SD	6.7	6	4	5	6	-	1	1	2	1	-
27	F	65	Adeno	N	IV	SD	9.8	1	-	-	1	-	0	-	-	0	-
29	F	65	Adeno	N	IV	PR	5.7	8	-	-	9	9	0	-	-	0	0

Dashes indicate that plasma was not available.

Abbreviations: F, female; M, male; Adeno, adenocarcinoma; Y, Yes; N, No;

PR, partial remission; SD, stable disease;

C3,taking during the third months in TKI treatment;

C4, taking during the fourth months in TKI treatment; PD, progression of disease;

PD6, six months after disease progression.

**Figure 4:**
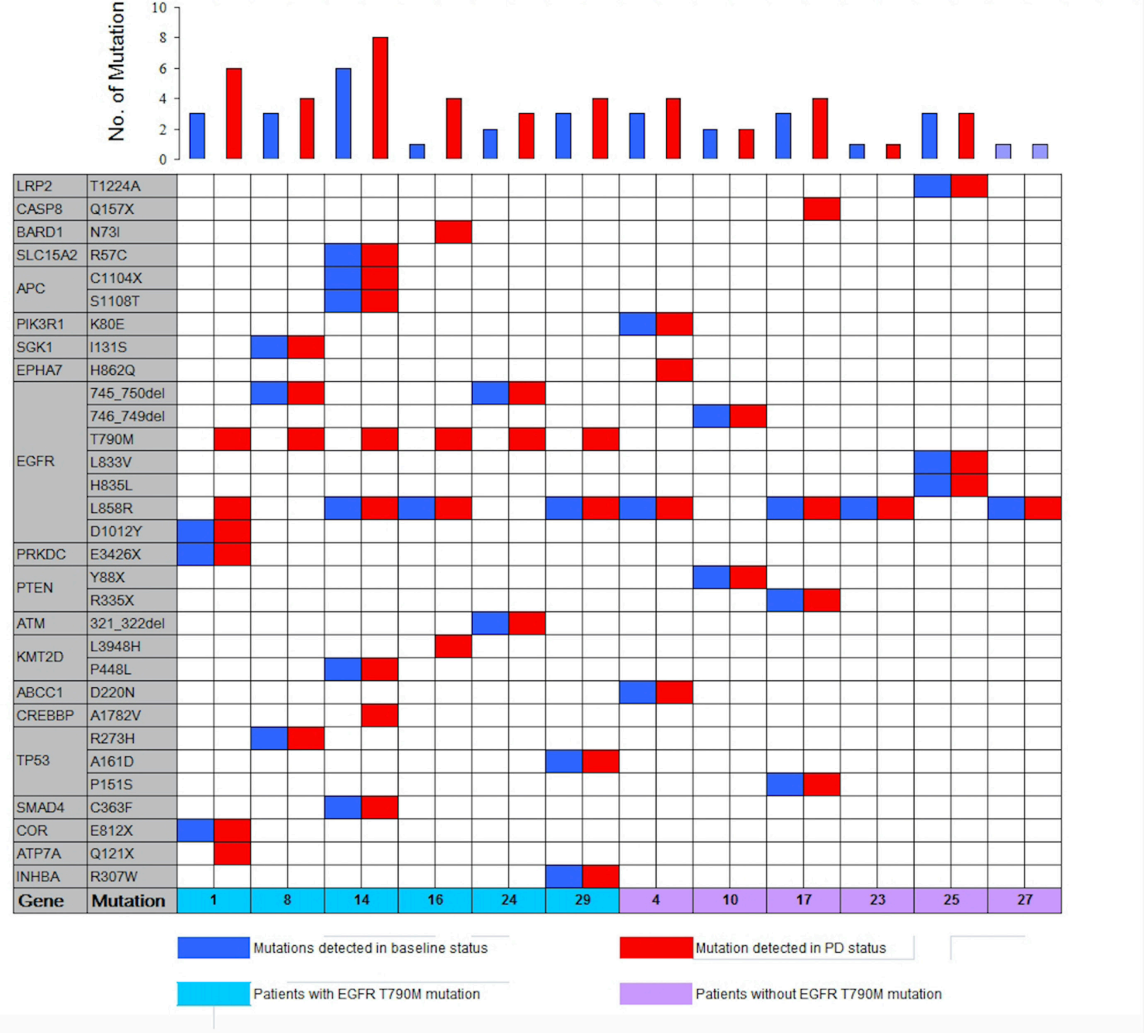
Heat map of meaningful mutant genes in ctDNA from 12 patients using next generation sequencing

**Figure 5:**
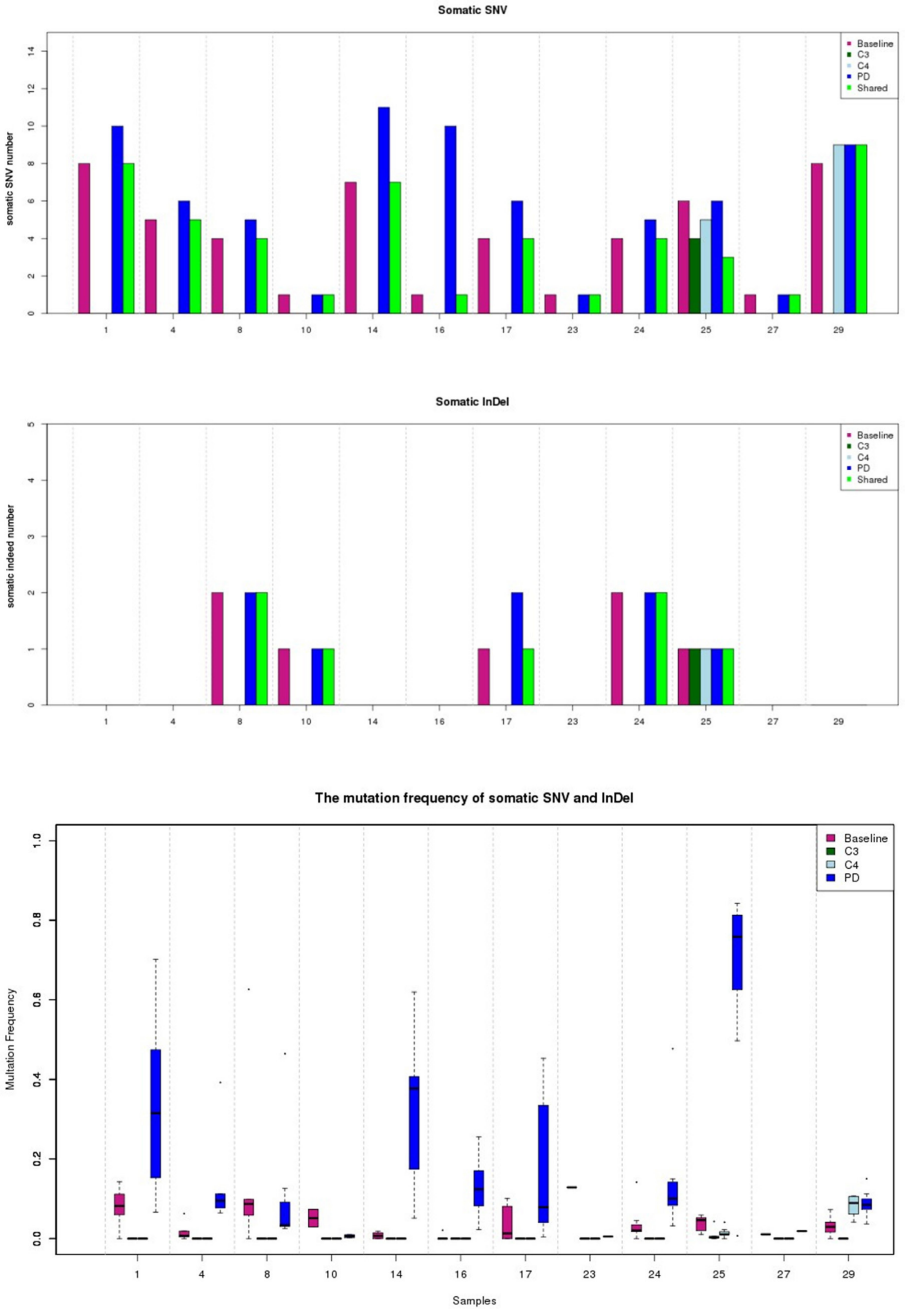
Dynamic changes of total mutational copies and frequencies in ctDNA detected by NGS Abbreviation: Baseline, plasma sampling before TKI treatment; C3, plasma sampling during the third months in TKI treatment; C4, plasma sampling during the fourth months in TKI treatment; PD, plasma sampling at the time of disease progression.

Using this approach, EGFR sensitive mutations were identified as the most common somatic variation in baseline plasma DNA; there were 3 cases with exon 19 deletions, 8 cases with the L858R mutation, and 1 case with overlapping L833V and H835L mutations. The T790M mutation was not identified in baseline plasma DNA. However, it was found in 6 of 12 patients (50%) in PD, and 5 of 6 T790M positive patients coupled with EGFR level increasing (Figure 4). In addition, 66.6% (8/12)of patients presented elevated frequencies of total mutational copy numbers, and 76.5% (26/34) of cases exhibited increased mutual mutation frequencies after PD (Figure 5).

**Figure 6:**
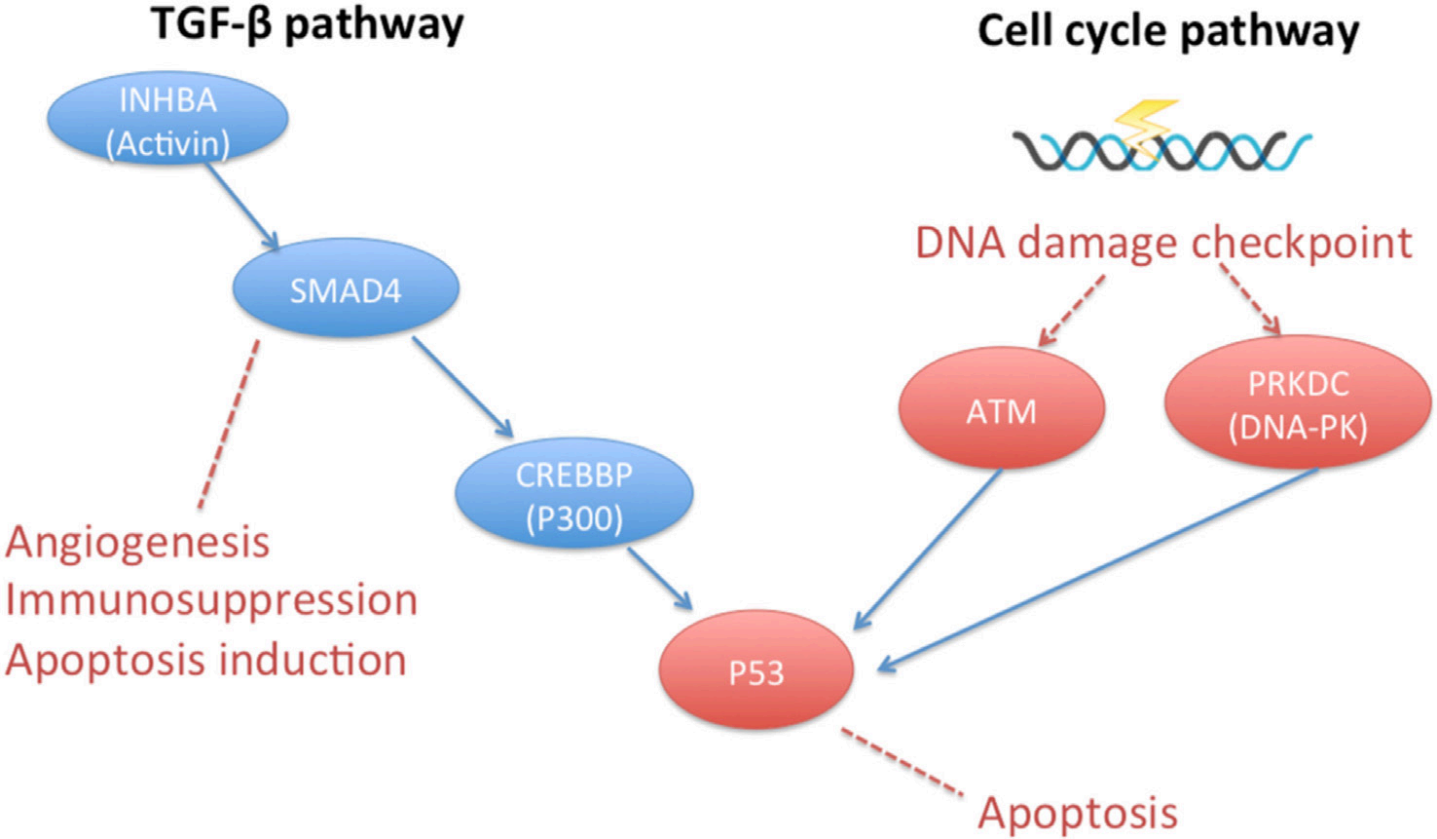
Signaling pathways detected by NGS Mutations involved in DNA damage checkpoint were shown in red, and mutations involved with TGF-β pathway were shown in blue.

Other mutations were identified as follows: 4 cases (33.3%, 4/12) with the TP53 mutation, 2 cases (16.7%, 2/12) with the PTEN mutation, and 2 cases (16.7%, 2/12) with the MLL2 (mixed-lineage leukemia 2) mutation. We further performed pathway analysis to identify other signaling cascades in addition to the canonical EGFR pathway. Mutated genes were remarkably enriched in a DNA damage checkpoint pathway that forms a part of the cell-cycle pathway. We also observed significant enrichment of mutated genes in TGF-β signaling in two cases that were synchronously accompanied by the T790M mutation (Figure 6). Most of the mutant genes existed during both baseline readings and during PD. An exception to this was the CREBBP mutation, which emerged in the course of PD. All of the mutation frequencies existing at both baseline and PD that were enriched in the DNA damage checkpoint pathway and in TGF-β signaling pathway increased in the course of PD.

### Comparison of the performance of ddPCR and NGS in ctDNA testing

**Figure 7:**
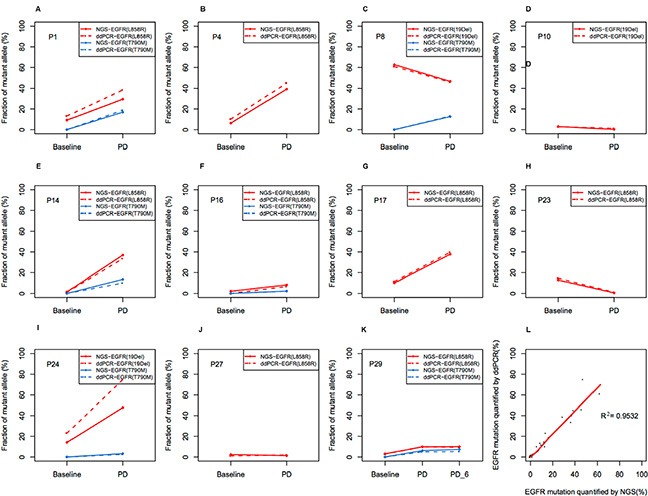
Comparison between NGS and ddPCR in measuring relative abundance of EGFR mutation in ctDNA (**A–J**) Allele frequency (AF) of EGFR mutations in each patient. The dashed line indicates AF measured by ddPCR, and the solid line indicates AF measured by NGS. EGFR mutation was shown in red line, while T790M was shown in blue line. PD, progressive disease; (**K**) Concordance between NGS and ddPCR in measuring relative abundance of EGFR mutation in ctDNA (*R*^2^ = 0.953).

We compared ddPCR with NGS methods for the analysis of 12 patients’ plasma samples. As mentioned above, the limit of quantitation for the ddPCR and NGS methods were 0.04% and 5%, respectively. For patients who had mutant frequencies equal to or greater than 5% in at least one plasma sample, both the sensitivity and the specificity of the NGS method were 100%. For patients who had mutant frequencies less than 5% in all of the plasma samples, some true positive mutations were filtered out by Perlscript in order to avoid excessive amounts of false positives. The sensitivity and specificity for NGS were 89% and 100% respectively. ctDNA levels of the same patient detected by both the ddPCR and NGS methods showed a similar trend. In general, quantification of mutant frequencies via the ddPCR and NGS methods showed excellent agreement (Spearman Correlation, *R*^2^ = 0.953, *P* < 0.001, Figure 7). These results validated the feasibility of both methods.

## DISCUSSION

Due to the existence of intra-tumor heterogeneity and dynamic alterations of EGFR-TKIs-related sensitive and resistant genes that occur in the process of therapy, the real-time, quantitative, and parallel detection of the status of multiple driver genes has value for evaluating suitable therapeutic regimens for EGFR-mutated patients. Here, our results indicated that ddPCR is a highly sensitive method for EGFR mutation analysis with ctDNA for advanced lung adenocarcinoma patients, with a detection limit 0.04%. Patients with high EGFR mutant abundance (> 5.15%) had a longer PFS as compared with those with low EGFR mutant abundances (≤ 5.15%). Dynamic changes in the abundance of EGFR mutations were associated with the survival of EGFR-TKIs treatment. NGS shows good performance for the testing of multiple genes simultaneously.

Previous studies have compared the coincidence rate between for the detection of EGFR mutations from ctDNA and tissue samples. ctDNA test sensitivity was 46–82%, specificity was 90–99%, and concordance was 78–88% in assessing EGFR mutation [11, 21–23, 32, 33]. Taking the detection of EGFR mutations in tumor tissue as the golden standard, the concordance of the detection of EGFR mutations from ctDNA was 74% (54/73)in our study. There was 26% (19/73) discordance between the tissue and plasma samples in our study with regard to the detection of EGFR mutations. A possible reason for this discrepancy may be that nearly 50% (36/73) of the patients in our study received EGFR-TKIs as the second (or beyond) line therapy, and the time-points for the collection of their blood specimens was different from that of the tissues samples, which were sampled before initial therapy. Chemotherapy prior to EGFR-TKIs may reduce EGFR mutation frequency in ctDNA of patients with advanced NSCLC [34].

In accordance with previous reports, our study showed that patients with EGFR mutations in TKI-naïve plasma had longer PFS and OS as compared to patients without EGFR mutations in plasma [11, 22, 23]. Further, we realized that, in addition to mutation status, the abundance of mutant alleles also predicted survival. In a previous study, Zhou *et al.* [35] demonstrated that the relative abundances of EGFR mutations in tumor samples could predict the potential benefit from EGFR-TKIs treatment for advanced NSCLC. However, the underlying biology of the quantitative analysis of EGFR mutations in plasma is not yet fully understood. Yung *et al.* [20] were the first to use digital PCR to quantify EGFR mutations in plasma and proved the relationship between the response rate and the abundance of EGFR mutations, but they did not analyze the predictive and/or prognostic value of ctDNA. Our results indicate that patients with highly abundant EGFR mutations (> 5.15%) at baseline had a longer PFS compared with those carrying low abundance EGFR mutations (≤ 5.15%). The relatively high frequency of intratumor EGFR mutant alleles might imply that TKIs-sensitive mutant clones accounted for the major part of whole tumor clones, and needed longer time to develop drug resistance compared with the those with low frequency of intratumor EGFR mutant alleles. Our previous [36] and other studies [35] have demonstrated that high frequency of EGFR mutant alleles yielded superior efficacy and survival of EGFR-TKIs compared with low frequency ones. This intra-tumor heterogeneity of EGFR mutation can be reflected by the quantification of EGFR mutant alleles in ctDNA detected by ddPCR. Therefore, we speculated that the superior survival of EGFR-TKIs therapy for patients with high quantities of EGFR mutations at baseline might be attributed to the presence of high percentage of EGFR mutant clones in the tumor entities.

Moreover, we found that patients undergoing decreasing EGFR mutation alleles had better survival compared with those without decreasing. The possible reason included that 1) the significant reduction of EGFR mutation in peripheral blood during therapy meant higher sensitivity of tumor to EGFR-TKIs therapy compared to those without decrease 2) EGFR mutant clones might also carry other gene aberrances, causing the activation of related pathways correspondingly and leading to resistance to EGFR-TKIs subsequently.

Using the NGS technique, somatic mutations such as PTEN, MLL2, and TP53 were also detected. Previous studies have suggested that PTEN loss contributes to erlotinib resistance in EGFR-mutant lung cancer via the activation of AKT and EGFR [37]. In this study, somatic mutations in PTEN were found in two patients. According to previous studies, the incidence of PTEN mutations in NSCLC is 4%–4.5%, and is more common in smokers and squamous carcinoma [38–40]. However, the PTEN mutation rate was 16.7% (2/12) in our study, and these were both detected in non-smoking women with adenocarcinoma. This result is similar to a study that reported a PTEN mutation rate of 16.1% in EGFR-mutant NSCLC [41]. Reasons for the differences might include the limited sample size or variations in different human sub-populations. We also found frequent mutation of the MLL2 gene in 2 EGFR-mutant patients. This gene encodes a histone lysine methyltransferase that plays an important role in the regulation of transcription. Our results were in agreement with a previous report in which deleterious mutations of MLL2 occurred in 11.4% of Chinese NSCLC patients [42].

In addition to the canonical EGFR pathway, we also found significant enrichment of gene mutations related with cell cycle pathway and the TGF-β pathways. TGF-β signaling pathway has been justified being associated with EGFR-TKIs resistance, which may be induced by epithelial-mesenchymal transition (EMT), the activation of MAPK pathway and IL-6 axis [43–46]. During the disease progression along with the enlarged tumor burden, rapid replication of cancer cell DNA produced more errors in the process of cell cycle, causing the activation of DNA damage checkpoint pathway especially in the present of dysfunctional mutations in TP53 and ATM. Previous studies have showed multiple mechanisms of resistance can occur synchronously in the same patient [43]. In this study, two cases harbored T790M together with the TGF-β pathway related genes variants, a situation that might indicate multiple mechanisms of resistance.

We used both ddPCR and NGS to monitor dynamic changes of EGFR mutation in ctDNA. Levels of EGFR-sensitive mutations and the T790M mutation analyzed via the two separate methods were significantly correlated with each other. The detection limit of ddPCR is about 0.04%, which is more sensitive and with lower cost than regular NGS analyses. However, one-time ddPCR assay can detect only one known gene mutation, while NGS can monitor multiple gene mutation simultaneously and identify novel functional gene aberrances in whole-genomic scope. So, as a quantitative detection method, ddPCR can be well applied to testing a known gene mutation, especially with low frequency.

There were several limitations to out study. This was a retrospective and single-institution study. Repeat biopsies were not performed to obtain matched tissue samples with blood after disease progression to confirm the presence of resistance related gene mutations. However, based on the positive and meaningful results in this study, we launched a multicenter clinical trial using ddPCR as a screening tool to select EGFR mutation patients in ctDNA to receive EGFR-TKI therapy (BENEFIT, NCT02282267), which had finished enrollment to date. This prospective multicenter study will further validate the conclusion of the present study.

In summary, plasma DNA testing is promising and should be ready for use in the near future for the quantitative and qualitative analysis of multi-gene variations using ddPCR and NGS. Highly abundant EGFR mutations in *de novo* plasma samples predicted longer PFS on EGFR-TKIs. Compared to the non-decreasing quantity group, patients with decreasing quantities of EGFR mutated alleles after PD showed better PFS and OS. Our results warrant further investigation in a prospective study.

## MATERIALS AND METHODS

### Sample collection and processing

Seventy-three patients diagnosed with advanced (stage IV) NSCLC admitted to the Peking University Cancer Hospital were recruited from May 1st, 2010, to April 1st, 2013. Enrollment Criteria included: 1) histological type must be adenocarcinoma; 2) patients were undergoing EGFR-TKIs treatment. 3) Patients’ tumor tissues carried EGFR mutations as detected by denaturing high-performance liquid chromatography (DHPLC).

DNA was extracted from 2 mL of plasma using QIAamp Circulating Nucleic Acid Kits (Qiagen) and eluted with 55 μl of Elution Buffer.

The study was approved by the Institutional Review Board of the Peking University Cancer Hospital. All of the patients signed informed consent before the collection and use of their tumor specimens and blood samples.

### Droplet digital PCR analysis for plasma samples

Droplet digital PCR assays were done using a QX100 Droplet Digital PCR System (BIO-RAD). The QX100 droplet generator was used to create an emulsion of ∼20,000 droplets, with mutant and wild type DNA distributed among the droplets following the Poisson distribution. After the PCR reaction, each droplet produced a positive or negative fluorescence signal indicating whether or not the EGFR mutant gene was present, respectively, in a given droplet. We then measured the absolute quantities of circulating EGFR mutant and wild-type alleles. The ratio of the concentration of mutant EGFR ctDNA to wild-type ctDNA was determined by ddPCR.

To evaluate the sensitivity of the ddPCR assays for mutant sequence detection, DNA extracted from cell lines carrying the L858R mutation, the T790M mutation, and exon 19 deletion (ATCC NCI-H1975, NCI-H1975 and NCI-H1650, respectively) were mixed with human reference genomic DNA (Promega) to achieve decreasing ratios (1:1 to 1:10,000) of the mutant allele versus the wild-type allele. Genomic DNA from the H1975 cell line expresses both the EGFR L858R and the T790M mutations. The three assays were able to detect the mutant alleles in samples containing 2,500 alleles, and the limit of quantitation was approximately 0.04%. Human reference genomic DNA and deionized water were used as negative controls. Mutant alleles from the NCI-H1975 and NCI-H1650 cell lines were used as positive controls [47].

### NGS-Library preparation, hybrid capture, and sequencing

DNA libraries with ∼150 nucleotide base pair insert size were prepared using a NEBNext DNA Library Prep Reagent Set (New England BioLabs), according to the manufacturer's protocol. Next, the libraries were hybrid-captured with custom biotinylated DNA oligo pools (custom SureSelect kit, Agilent) that contained all of the exons of 483 cancer-related genes (driver genes from the Catalogue of Somatic Mutations in Cancer, COSMIC V68) and 88 introns from 14 genes that are frequently rearranged in cancer. All samples were sequenced with the Illumina HiSeq2500 platform in a 150 bp paired-end pattern.

### Calculation of absolute and relative EGFR mutant allele quantities by ddPCR

As described in a previous paper [47], the absolute and relative quantities of EGFR mutated alleles were determined by analyzing the number of positive and negative fluorescence signals in droplets. Plasma sample DNA input, as a unit of ‘genome copies per reaction’ represented the absolute abundance of EGFR mutations. The percentage of EGFR mutated alleles represented the relative abundance of EGFR mutations.

### Statistical analysis

We used SPSS statistical software, version 17.0 (SPSS), to analyze the data. We used χ^2^ tests to assess the relationships between EGFR mutation status and clinicopathologic features. Median PFS (progression free survival) and OS (overall survival) were calculated by Kaplan-Meier estimation. Log-rank tests were performed to explore the cut-off values of the quantity of EGFR mutated alleles that could predict efficacy outcomes. *P* < 0.05 was considered significant. Relationship between metastatic sites (reflecting tumor burden) and previous treatment with ctDNA levels by Kruskal-Wallis Test. DAVID software (Database for Annotation, Visualization, and Integrated Discovery) was used to analyze cancer genes in pathway enrichment.
